# Bioremediation of Heavy Metals and Organic Toxicants by Composting

**DOI:** 10.1100/tsw.2002.91

**Published:** 2002-02-12

**Authors:** Allen V. Barker, Gretchen M. Bryson

**Affiliations:** Department of Plant and Soil Sciences, University of Massachusetts, Amherst, MA 01003, USA

**Keywords:** cocomposting, pesticides, pesticide degradation, xenobiotics, heavy metals, polycyclic aromatic hydrocarbons, polychlorinated biphenyls

## Abstract

Hazardous organic and metallic residues or by-products can enter into plants, soils, and sediments from processes associated with domestic, municipal, agricultural, industrial, and military activities. Handling, ingestion, application to land or other distributions of the contaminated materials into the environment might render harm to humans, livestock, wildlife, crops, or native plants. Considerable remediation of the hazardous wastes or contaminated plants, soils, and sediments can be accomplished by composting. High microbial diversity and activity during composting, due to the abundance of substrates in feedstocks, promotes degradation of xenobiotic organic compounds, such as pesticides, polycyclic aromatic hydrocarbons (PAHs), and polychlorinated biphenyls (PCBs). For composting of contaminated soils, noncontaminated organic matter should be cocomposted with the soils. Metallic pollutants are not degraded during composting but may be converted into organic combinations that have less bioavailability than mineral combinations of the metals. Degradation of organic contaminants in soils is facilitated by addition of composted or raw organic matter, thereby increasing the substrate levels for cometabolism of the contaminants. Similar to the composting of soils in vessels or piles, the on-site addition of organic matter to soils (sheet composting) accelerates degradation of organic pollutants and binds metallic pollutants. Recalcitrant materials, such as organochlorines, may not undergo degradation in composts or in soils, and the effects of forming organic complexes with metallic pollutants may be nonpermanent or short lived. The general conclusion is, however, that composting degrades or binds pollutants to innocuous levels or into innocuous compounds in the finished product.

## INTRODUCTION

Hazardous organic materials are used or are generated widely in domestic, municipal, agricultural, industrial, and military activities. From agricultural, home, and industrial usage, pesticides can enter into crop residues, municipal sludges, farm manures, and soils[1]. Insect, disease, and weed control in farming may lead to accumulation of pesticides in soils, in which decomposition of these materials may occur slowly[2,3,4]. Metallic contaminants in organic materials and land arise from pesticides and from farm manures[5,6,7,8,9]. Major inputs of metals into the environment also occur from municipal and industrial sludges and by-products[10]. Pesticides and metals in feedstocks (materials that are composted) or in finished composts may be hazardous to people who handle the materials, may enter into the environment, and may be harmful to plants growing in compost-amended soil, to soil biota, or to livestock that may eat the feedstock, compost, or plants grown on compost[9,11,12,13,14,15,16,17,18,19,21,22,23,24]. In recent times, however, by controlling the quality of feedstock used in composting, the metals contents of composts have been reduced relative to those of the past. Analysis of selected European composts showed that the majority were high quality with regards to low metallic composition with the possible exception of cadmium, which was still far below U.S. federal standards[25,26].

Shimp[27] noted the requirements for evaluating the safety of compost-borne materials; among these criteria were the concentration of contaminating ingredients in the compost and concentrations that might cause harm in the environment. Environmental concerns include phytotoxicity, food-chain safety, and ingestion safety, among others[28]. Phytotoxicity of biodegradation products from inadequately cured composts, general and specific soluble salts, and plant nutrient immobilization are short-term problems that may arise as a result of use of composts[16].

Organic contaminants (e.g., polychlorinated biphenyls [PCBs], polycyclic aromatic hydrocarbons [PAHs]) other than pesticides can enter into the soil from fuel combustion or from sewage sludge and other feedstocks[29,30,31]. These contaminants from sludge additions to land may persist in soils or contaminate forage on which livestock graze[32]. Spillage of fuel oil hydrocarbons can contaminate soils. These hydrocarbons will inhibit seed germination and plant growth, but plants do not appear to accumulate the hydrocarbons[33].

Military activities result in deposition of explosives or components of explosives in land[34]. Perchlorates are constituents of propellants, explosives, and military batteries and are deposited on land[35]. Perchlorates are water-soluble contaminants and may enter water supplies or be absorbed by plants, thereby having direct effects on human health[36].

Composting is applied in bioremediation as a means of degrading toxic organic compounds and perhaps lessening the toxicity of metallic contaminants in organic residues, wastes, and by-products[37,38,39,40,41]. In addition, composting stabilizes wastes for ultimate disposal in traditional manners in landfills or on farmland[42].

Composting has been practiced to reduce volume and water content of feedstock, to destroy pathogens, and to destroy odor-producing nitrogenous and sulfurous compounds[43,44]. Nitrate leaching can occur from unlined landfills[45]. Composting of nitrogen-rich materials with other feedstocks may lead to stable materials less likely to contaminate water than will the addition of the nitrogen-rich material alone to land or to landfills.

Composting also is considered as a remediation method for handling contaminated soil, sediment, and organic wastes[34]. Mechanical treatment by grinding, mixing, and sieving out nondegradable or disturbing materials (metals, plastics, glass, stones) gives good conditions for biological treatment of compostable materials[46]. The biological treatment builds up stable organic compounds through humification and reduces concentrations of organic pollutants. Composting can be used to lower the levels of chemical contaminants in residues or in soils to which polluted residues have been added.

The processes of remediation in compost are similar to those that occur biologically in soil. However, composting may accelerate the destruction of contaminants[15,34,47,48]. Temperatures are generally higher in composts than in soils, resulting in increased solubility of contaminants and higher metabolic activity in composts. High levels of substrate in composts can lead to cometabolism of organic contaminants. The microbiological population can be more numerous and diverse in composts than in soils. The nature of the organic contaminant, composting conditions and procedures, microbial communities, and time all affect mechanisms of conversions in composts or soils[15,34,47,48,49].

## FATE OF ORGANIC SUBSTANCES DURING COMPOSTING

Modern pesticides (insecticides, fungicides, herbicides) are mostly organic compounds that are subject to biological decomposition. Pesticides added to soil can persist for a year or more[50,51]. Sometimes, the lack of sufficient readily decomposable organic matter in soil gives inadequate substrate to stimulate microorganisms in the decomposition of pesticides[50,52]. Simazine (6-chloro-N’, N’-diethyl-1,3,5-triazine-2,4-diamine) remained in soil for 4 years after application[53]. Carbofuran (2,3-dihydro-2,2-dimethyl-7-benzofuranyl methylcarbamate) was more labile than simazine, but residual carbofuran that had become bound to soil was more recalcitrant to degradation than freshly added pesticide[54]. Tillage practices did not affect the dissipation of herbicides in farmland[55,56,57]. The vigorous biological activity during composting can be used to accelerate or enhance the decomposition of pesticides in soil or deliberately to treat pesticide-contaminated materials[15].

Büyüksönmez et al.[15] reviewed the occurrence and fates of pesticides in feedstock and in composts. Generally, investigations detected few targeted pesticides in either feedstock or in finished compost. On the other hand, Rynk[58] reported that herbicide contamination in composts made from lawn clippings or pasture grasses was suspected of damaging production of greenhouse crops. Composting can degrade various organic compounds that might be present in feedstock, if the process is carried out with proper aeration, water, C:N ratios, and duration[59]. Strom[60] noted that pesticide levels in composts were low, partly because of low initial contamination of feedstock and because of degradation of pesticides during composting. Chaney et al.[61] and Vogtmann and Fricke[62] noted that either mature or immature composts are generally low in xenobiotic organic compounds, suggesting that degradation of pesticides occurs early in the process. Composting typically proceeds in three phases[63]: a rapid decomposition phase of about 30-days duration, followed by a stabilization phase of about 50 days duration, and a maturation phase following the stabilization phase. Rapid degradation of xenobiotics commonly occurs during the first 30 days. Some xenobiotics that resemble aromatic substances in mature compost might be recalcitrant to decay.

Büyüksönmez et al.[15] noted that the majority of the pesticides detected in feedstocks or composts were organochlorine insecticides, which were resistant to biodegradation and included some banned in the U.S. Strom[60] noted detectable levels of chlordane (a polychlorinated hydrocarbon, methanoindine derivative) in yard trimmings but no detectable level of 26 other pesticides. Organophosphate and carbamate insecticides and most herbicides were detected rarely and, if present, decomposed during composting. The Büyüksönmez review[15] noted that mineralization of organic pesticides was only a small fraction of pesticide degradation, with other prominent fates of the pesticides being partial degradation to secondary compounds, adsorption to compost, and volatilization.

The effects of composting on pesticides is not always favorable. The secondary compounds may be as, or more, toxic than the original pesticide. Losses by volatilization essentially mean that the pesticide has been moved from one place to another. If recalcitrant materials, including metals, are present in feedstock, they may be unchanged or concentrated during composting. Büyüksönmez et al.[15] presented a comprehensive review in which a number of pesticides are classified by use and by persistence. Persistence is a relative term for qualitative assessment of pesticide degradation. Half-life, the number of days needed for a pesticide to decompose to half its original concentration, is a term used commonly to express persistence. Pesticides with a half-life of 30 days or less are considered nonpersistent, and those with a half-life exceeding 100 days are considered persistent[15]. Recalcitrant pesticides are persistent and may show no changes during composting or have a half-life of a year or more. Other reviews providing discussions of pesticide and organic compound degradation during composting include Fogarty and Tuovinen[64] and Epstein[65].

Destruction of pesticides depends on the pesticide and on the substrate in which the pesticide is undergoing cocomposting. In composting of food wastes, diazinon [O-O-diethyl-O-(2-isopropyl-6-methyl-5-pyrimidinyl) phosphorothionate] and parathion (O-O-diethyl-O-4-nitrophenyl phosophorothionate) were destroyed rapidly and almost completely; dieldrin (a polychlorinated dimethanonaphthalene) was reduced substantially, but DDT (dichlorodiphenyltrichloroethane) was recalcitrant[66]. Residues of DDT also persist for lengthy periods in soils[67]. Benzo(a)pyrene (a PAH) persisted in composting of municipal trash[68]. Carbaryl (1-naphthyl N-methylcarbamate) and phenanthrene (a PAH) persisted in composting of sewage sludge in benchtop experiments. Mineralization of atrazine [6-chloro-N-ethyl-N’-(1-methylethyl)-1,3,5-triazine-2,4-diamine] was low during composting of atrazine-amended poplar wood (*Populus* sp. L.) or corn cobs (*Zea mays* L.) at three temperatures, suggesting no relationship between atrazine and mineralization of these lignocellulosic substrates[48]; however, mineralization of atrazine was enhanced by cocomposting with newsprint[47]. Composting destroyed nearly half of the 2,4-D (2,4-dichlorophenoxyacetic acid) residues of lawn clippings[69]. Composting of contaminated feedstocks of mixed broadleaf tree leaves and grass clippings converted diazinon {O,O-diethyl O-[6-methyl-2-(1-methylethyl)-4-pyrimidinyl] phosphorothionate} to less phytotoxic metabolites or to other residues with low bioavailability, although the fraction of mineralization of total diazinon to carbon dioxide was much smaller (11%) than mineralization of total organic carbon (48%) in the feedstock[70]. Degradation of insecticide and herbicide residues to low or nondetectable levels occurred in grass clippings taken from test plots of sprayed turfgrass and stored or composted for 21 days[71]. After one year of composting of grass clippings, herbicide residues were reduced to concentrations at or below levels of detection[72]. In laboratory composting of biosolids and wood chip feedstocks over an 18- to 20-day period, carbaryl was degraded to 3 to 4% of its original concentration, but phenanthrene persisted at 89 to 93% of its original concentration[73]. Rynk[58] noted that picloram (4-amino-3,5,6-trichloro-2-pyridinecarboxylic acid) (half-life of 20 to 300 days, averaging about 90 days) contamination was responsible for injuring plants in one instance, and that clopyralid (3,6-dichloro-2-pyridinecarboxylic acid) (half-life of 40 to 70 days) was responsible in another case. The general conclusion from research with pesticide degradation by composting was that pesticide concentrations were lowered to nonhazardous levels for crops in soils receiving the composts. However, if pesticides that are persistent or recalcitrant to degradation are present in the feedstock, special care may be needed not to use the compost on sensitive crops in the year in which the compost was made. Caution may need to be taken in some cases not to grow plants in 100% compost in containers.

The composting of wood containing PAHs was studied under laboratory conditions[74]. Wood was artificially contaminated with PAHs (phenanthrene and pyrene) or was obtained from preservative-treated wood (such as chipped railroad ties). The wood was soaked with liquid hog manure before composting. After 61 days of composting in Dewar vessels, PAH concentrations in the artificially contaminated wood were reduced from 1,000 mg/kg of each PAH to 26 mg phenanthrene/kg and to 83 mg pyrene/kg. For the preservative-treated wood, the degradation of PAHs was somewhat slower than with the artificially contaminated wood; this result was attributed to the presence of more complex, condensed types of PAHs. Adding liquid manure to the compost mix accelerated temperature rises in the vessels and evolution of carbon dioxide relative to wood receiving no supplement or to wood receiving a source of mineral nitrogen and other nutrients. Adding compost, decomposed wood, or hydrocarbon-contaminated soil also enhanced rises in temperature and evolution of carbon dioxide relative to the results with untreated wood.

A study by Raymond et al.[75] demonstrated that paraffin wax–coated corrugated cardboard decomposed well during composting, with most of the wax coating being decomposed during 12 weeks of composting. Composting also has potential for destruction of compounds such as acrylamide, for which decomposition in soil has been shown to be accelerated by nitrogen additions and increased temperatures such as those that might occur in compost piles[76].

Cutting and composting may be a way of controlling the allelopathic effects of certain weeds on beneficial plants. Composting (55 days or longer) of leaves of *Eupatorium adenophorum* Spreng. and *Lantana camara* L., which are allelopathic to crops, eliminated the allelopathic effects of the residues on *Brassica rapa* L. (turnip) and reduced such effects on *Sorghum vulgare* Pers. (grain sorghum)[77].

Composting of contaminated soils can convert hazardous wastes into innocuous end products[78]. Williams and Keehan[34] reviewed and reported research on destruction of explosives (trinitrotoluene, nitrocellulose, and others) during composting of soils and organic matter. They concluded that explosives are reduced to low concentrations during composting. Conversions of perchlorate to innocuous compounds, such as chloride, have been attempted in bed reactors[79]. Bacterial degradation of perchlorate in water has been demonstrated[80]. Composting should be an excellent means for decontaminating perchlorate-polluted soils.

Windrow composting of soil contaminated with 212 mg/kg chlorophenols (dry mass basis) from wood-preserving facilities allowed for degradation of the pollutant to levels of about 30 mg/kg in 4 months[81]. Pilot studies in laboratory experiments showed that degradation was microbially mediated and that volatilization losses of chlorophenols did not exceed 1.5% of the total initial contamination. A second summer season of composting led to little further decrease in chlorophenol concentrations in the soil.

Fuel oil hydrocarbons are inhibitory to plant growth[33]. Beaudin et al.[82] studied the composting of hydrocarbon-contaminated soil with alfalfa and maple leaves. For soils contaminated with mineral oils and grease (by mass, 40% aliphatic, 32% polar, and 28% aromatic hydrocarbons) at 17,000 mg total hydrocarbons per kilogram, 60% of the aliphatic, 54% of the aromatic, and 83% of the polar compounds had degraded in 180 days of composting. This result compared with only 50% degradation of mineral oils and grease in land. After 287 days of composting, 73% of all mineral oils and grease had degraded. Englert et al.[83] reported that the cost of composting of contaminated soils in piles is only slightly more expensive than land farming. Biodegradation of fuel oil hydrocarbons in soil was increased by heating and forced aeration of soils[84].

Composts may be used as biofilters to cleanse air of pollutants. For example, finished compost was used to support microbial degradation of trichloroethylene (TCE)[85]. Up to 99% of the TCE in headspace above compost was removed after 96 h of exposure with compost enriched with propane or methane to stimulate cometabolism of TCE.

## FATE OF INORGANIC SUBSTANCES DURING COMPOSTING

Metallic contaminants may enter composts from farm manures[9,86], sewage sludges[87,88], municipal solid wastes[16,89], and pesticides[90]. Unlike organic contaminants, metallic contaminants in feedstock are not lessened quantitatively during composting; however, the bioavailability of metallic contaminants may be lessened by composting[16]. Humic substances and iron oxides in composts can limit the bioavailability of metals as well as organics in composts.

Because of the loss of carbon during composting, the concentrations of metallic contaminants in solid wastes may be increased. Paré et al.[91] reported increases in total concentrations of some residual heavy metals, ranging from 26 to 145% increases after 41 days of composting of biosolids and municipal solid wastes; concentrations of some other metals remained constant or decreased during composting. Sequential extraction of metals indicated a decline in soluble components and an increase in residual, organically bound forms[91]. Paré et al.[92] determined that amounts of water-soluble and acid-hydrolyzable fractions of carbon and nitrogen compounds declined during composting, that nonhydrolyzable carbon compounds remained somewhat constant, but that nonhydrolyzable nitrogen-containing compounds greatly increased. Paré et al.[91,92] suggested that the stabilized organic matter formed complexes with metals and restricted the mobility of the metals and their availability for plant absorption. However, sometimes, as in nature, composting may release contaminating metals from organic combination by degradation of the organometallic complexes, leading to increased bioavailability of metals[93].

Diets of poultry and livestock include metals and metalloids (As, Co, Cu, Fe, Mn, Se, Zn) to prevent diseases, improve weight gains, and increase egg production[8]. Feeding these elements to livestock and poultry leads to elevated concentrations in manure. Treatment of farm manures with aluminum sulfate lowered runoff of arsenic, copper, iron, and zinc from plots fertilized with poultry manure relative to runoff from plots fertilized with untreated manure[94]. Aluminum sulfate was also effective in restricting ammonia volatilization from poultry manure[95]. Perhaps treatment of poultry manure with aluminum sulfate will help to limit the availability of metals in contaminated compost and to restrict losses of ammonia during composting.

In raw farm manures and in composts, metals, such as copper, are associated with the organic fraction[96,97,98]. Hsu and Lo[5] reported that the leaching of copper, manganese, and zinc from composted hog *(Sus scrofa)* manure was low due to interactions of the metals with the organic material in the compost. Immature composts, however, had a higher potential for leaching of copper than mature composts. Some of this effect may have been pH-related, as extractable manganese and zinc were low in alkaline conditions. On the other hand, in alkaline-stabilized sludges, leaching of metals may be high, due to the dissolution of organic matter at high pH[99].

Cocomposting of sewage sludge and lime lowered extractable copper, manganese, zinc, and nickel relative to unlimed sewage sludge compost, although lime additions slowed composting[100]. A lime addition of 1% by mass was recommended for maximum effectiveness. Petruzzelli[101] and Ciavatti et al.[102] also reported that increasing the pH of compost lowered the availability of heavy metals.

Silicone polymers from industrial and consumer products may enter into municipal sewage sludge. A silicone polymer (polydimethylsiloxane) did not degrade during composting but did not appear to interfere with composting[103]. Silicone polymers are reported to degrade readily in soils[104].

## EFFECTS OF COMPOST ADDITIONS TO SOILS

Interest exists in the retention or degradation of pesticides in soils due to the potential for residual effects on subsequent crops, potential pesticide leaching to groundwater and streams, and common use of soils in container production of crops. Degradation of pesticides in soils may proceed slowly. Bioremediation by cocomposting is an economically attractive method for cleaning petroleum-contaminated or otherwise hydrocarbon-polluted soils. Cocomposting of hydrocarbon-contaminated wood products also has potential in environmental protection. Considerable research has been conducted to determine the fates of pesticides and other xenobiotic organic chemicals in composting and in the composting of similarly contaminated soils, wood products, and other residues. Research also has been conducted into the decomposition of composts in soils and the effects of their decomposition on mineralization of organic materials contained in the soil or in the compost[3,87,105,106,107,108].

Degradation of a fumigant (1,3-dichloropropene) in soil was enhanced in soils amended with compost compared to degradation in unamended soils[87]. A more mature, and hence more decomposed, compost was more effective in accelerating degradation than a less mature compost. A layer of compost on the surface of the soil lessened emission losses of the fumigant in relation to losses from a bare soil.

The principal effects of composts on activities of pesticides in soil follow the actions of composts on the chemistry and biology of the soil. Gevao et al.[3] reviewed the significance of bound pesticide residues in soils and covered, in part, the effects of organic matter and inorganic fertilizer additions on the fate of chemicals added to soils. The review noted that some research demonstrated enhanced dissipation and an increase in bound residues of organic chemicals following additions of organic matter to soils. Modifications of pesticide activity in soil depend on the nature and reactivity of the organic additions and their effects on microbial activities[105,106]. The capacity of a soil to degrade 2,4-D depends on its microbiological activity[107]. Topsoil with high organic matter content and hence more microbiological activity degraded 2,4-D more actively than subsoil. Mixing topsoil with subsoil increased 2,4-D degradation relative to that in the subsoil alone. Compost additions improved degradation of two herbicides [benthiocarb (S-4-chlorobenzyl diethylthiocarbamate) and MCPA (4-chloro-2-methylphenoxyacetic acid)] in soil[108]. However, additions of compost suppressed soil mineralization of atrazine (2-chloro-4-ethylamino-6-isopropylamino-*s*-triazine) relative to rates in unamended soils or in soils amended with starch or rice hulls[105]. The suppressive effect of compost on atrazine mineralization was attributed to the high nitrogen content of the compost, since all treatments receiving supplemental inorganic nitrogen fertilization also gave lower rates of atrazine mineralization than those without nitrogen addition; hence, in the presence of nitrogenous amendments, the nitrogen of the atrazine was less subject to mineralization[105]. Results with respect to the effects of nitrogenous amendments on mineralization of pesticides have varied widely, however.

If organic matter and nitrogen are limiting factors in mineralization, additions of compost might stimulate microbial activities and, consequently, accelerate pesticide degradation. This action was demonstrated with degradation of atrazine in soils amended with farm manures, straw, and nitrogen fertilizers[106]. On the other hand, organic matter additions, like compost incorporation into soil, may initially increase stability and decrease mineralization of pesticide residues by increasing soil organic matter and pesticide sorption to the organic matter[109]. Benefits may be accrued from sorption, as sorption of pesticides to organic matter generally restricts their leaching[110,111], but some organic amendments produce soluble organic compounds that enhance desorption and water solubility of pesticides[112]. Sorption of organic chemicals onto organic solids in soil might alter the degradation of the organic chemicals, as sorption may provide protection against microbial degradation, especially if desorption rates are slow. Benoit and Barriuso[113], using fresh wheat (*Triticum aestivum* L.) straw or straw compost to model humification of fresh organic matter, noted a decrease in mineralization of chlorophenols added with the straw or compost and an increase in formation of nonextractable or bound residues following incubation in soil. Benoit et al.[114] later reported, however, that protection of chemicals against biodegradation cannot be generalized, since sorption of chemicals onto straw compost before addition to the soil increased the rate of mineralization of the chemicals.

The application of raw or composted sewage sludge to soils generally increased organic carbon content and increased the retention of atrazine by sorption[115]. The effects varied with amount and kind of sludge applied. For example, limed sludge at low application rates diminished atrazine sorption relative to that measured for soil alone, but at higher rates the sorption effects of sludge organic matter dominated and atrazine retention increased. The effects of dissolved organic matter released from the sludge also varied with the kind of sludge applied. With raw sludges, the dissolved organic matter decreased atrazine sorption, whereas with composted sludge, atrazine sorption increased.

Addition of sewage sludge to soil enhanced degradation of the herbicides, 2,4-D and 2,4,5-T (2,4,5-trichlorophenoxyacetic acid)[116]. The degradation of the herbicides varied with soil type and whether the sludge was fresh or preconditioned (composted in soil). Fresh sludge additions had little effect on herbicide adsorption, but adding preconditioned sludge increased herbicide adsorption.

Conte et al.[117] studied the retention (recovery, lack of degradation) of PAHs in soils. Effects of addition of humic acids, such as might be released from compost or native soil organic matter, on the recovery of PAHs from soils were studied. Retention (recovery) depended on the specific nature of each PAH and increased with additions of humic substances. In soils where the native organic matter was destroyed by hydrogen peroxide, recoveries were higher than in soils in which the native organic matter was retained. This result may be due to differences in microbial populations in the oxidized or unoxidized soil as well as to the effects of native organic matter on stability of PAHs.

Fairbanks et al.[118] reported that sewage sludge additions to PCB-enriched soil suppressed PCB volatilization and increased the degradation of PCBs in soil. Mineralization was the principal route for loss of PCBs from sandy loams or clay evaluated in the study. Sludge addition to PCB-contaminated soil did not increase environmental hazards in any case. Brunner et al.[119] noted that degradation of PCBs in soil was enhanced by bacterial inoculation. The organism, *Acinetobacter*, was suggested as being superior to soil-borne microflora in the mineralization of PCBs.

In bench-scale experiments, Brown et al.[120] studied on-site treatment of contaminated soils that contained structurally complex petroleum compounds from an inactive oil refinery. Sawdust was added to all soils in the study except for one treatment, which involved only contaminated soil. Other treatments, or bulking agents, included a high-nitrogen sewage sludge compost and a low-nitrogen sewage sludge compost with sand. When used, the bulking agents constituted from 50 to 75% of the mixtures. In the soil with no amendments, only minor losses of n-alkanes occurred in 110 days, the duration of the study. Loss of n-alkanes, the most readily degradable petroleum hydrocarbons, was rapid in soil mixtures containing the high-nitrogen sludge compost but was slower in the mixtures with sawdust or low-nitrogen sewage compost. After 110 days, isoprenoids (pristane and phytane) had nearly disappeared from mixtures with the high-nitrogen sludge compost, and nearly half of the complex branched and cyclic alkanes had degraded.

Composting frequently generates aliphatic hydrocarbons of microbiological origin during the process[121]. Subsequent application of these compounds to land has been suggested to generate problems of phytotoxicity. Biological degradation in the soil generally alleviates any potential toxicity of compounds generated during composting, and the application of compost at agronomic rates should not deliver sufficient amounts of these compounds to cause phytotoxicity. Users, however, should allow sufficient lead time between applications of composts to land and planting of crops to avoid phytotoxicity.

Another factor to consider in the biological effects of composts on soils is the salinity of compost. Tester and Parr[122] reported that leaching of composts to remove salts, and also readily decomposable water-soluble carbon compounds, gave an initial lag in the decomposition of the compost, but with time, leaching accelerated decomposition where high rates of compost

(224 Mg/ha) had been applied. Nitrogen in unleached compost was mineralized, whereas in soils that received the leached compost, a net immobilization of nitrogen occurred. This result was associated by the authors with an increase in compost degradation in the soil.

Addition of crop residues or animal manures to selenate-treated soils restricted accumulation of selenium by canola (*Brassica napus* L.) or tall fescue (*Festuca arundinacea* L.)[11]. Land application of fly ash-organic waste mixtures to land resulted in an initial increase in soil solution concentrations of some metals (As, Se, Mo, Cu)[123], but the increases in specific metals varied in magnitude according to the kind of organic addition (poultry litter, sewage sludge). Within 30 days after application of the wastes, soil solution concentrations of metals from all treatments approached those in unamended soils. In a pot experiment, additions of cocomposted fly ash and lignocellulosic residues (yard and garden wastes) to soils generally increased bacterial and actinomycete counts but did not affect fungal populations[124]. Due to the increase in soil pH following the addition of either alkaline fly ash or the compost, plant uptake (*Vicia faba* L.) of heavy metals (Cd, Cr, Cu, Ni, Pb, Zn) was suppressed in a sandy soil but not in a clayey soil in which high buffering capacity mitigated the effects of fly ash amendments. Simeoni et al.[125] reported that lettuce (*Lactuca sativa* L.) and oat (*Avena sativa* L.) yields were higher in soil treated with sewage sludge compost than in soil treated with sewage sludge, especially at high rates of application (240 Mg/ha). In fact, yields were suppressed as sludge applications increased from 0 to 240 Mg/ha but were increased generally with increased compost applications. The yield differences were more notable in acid soil (pH 5.4) than in neutral soil (pH 7.6). Simeoni et al.[125] attributed the differences in yields to the slight liming effect of the compost raising the pH of the soils. Cadmium and zinc accumulation increased significantly more in plants grown with increasing sludge additions than in those with increasing compost additions. Increases were much greater in the acid soil than in the neutral soil.

Metals, such as copper, zinc, and cadmium, may be added to soil by applications of contaminated residues such as sewage sludge. Concerns exist that if applications of sludge are ceased after several years of application to land, mineralization of the sludge could release metals into soluble forms[126]. Perhaps maintaining organic matter at elevated concentrations imparted by sludge application might help to restrict phyotoavailability of metals. Hyun et al.[88] concluded, however, that phytoavailable cadmium did not increase as organic matter decreased in soils after sludge application was terminated.

Mobility of metals in compost varies with their speciation. Sawhney et al.[127] noted that leaching of arsenic (more than 20% of the initial arsenic content of compost) was much higher than leaching of cadmium, chromium, nickel, and lead (about 3% of the total initial content). Leaching of arsenic declined rapidly with time before reaching a baseline, indicating that the initial leaching was of water-soluble salts or exchangeable ions and that solid phases governed the subsequent slow-release processes. Compost standards usually refer to permissible levels of metals on a total basis of concentration. Leachable concentrations may be of more environmental importance than total concentrations. Profiles of fractions of metals in various combinations in composts differed widely according to metallic species[96].

## SUMMARY

Handling, ingestion, application to land, or other distributions of contaminated residues and by-products materials into the environment can render harm to humans, livestock, wildlife, crops, or native plants. Pollutants of pesticides, hydrocarbons, and metals may accumulate in plants, soils, and sediments. Considerable alleviation of hazardous wastes or contaminated plants, soils, and sediments can be accomplished by composting. Compostable substrates (feedstocks) contain metabolizable carbon, which will enhance microbial diversity and activity during composting and will promote degradation of xenobiotic organic compounds, such as pesticides, PAHs, and PCBs. In consequence, degradation of xenobiotic organics during composting is much faster than in unamended soils on site. Metallic pollutants are not degraded during composting but may be converted into organic species that are less bioavailable. Degradation of organic contaminants in soils is facilitated by addition of composted or raw organic matter, thereby increasing the substrate levels for cometabolism of the contaminants. Recalcitrant materials, such as organochlorines, may not undergo degradation in composts or in soils, and the effects of forming organic complexes with metallic pollutants may be nonpermanent or short lived. The general conclusion is, however, that composting degrades or binds pollutants to innocuous levels or into innocuous compounds and has substantial potential for remediation of polluted materials.

## BIOSKETCH

**Allen V. Barker**, Ph.D., is Professor, Plant & Soil Sciences, University of Massachusetts, Amherst. He received his Ph.D. in agronomy from Cornell University. Dr. Barker has been with the University of Massachusetts for 37 years. His research focuses on studies of plant nutrition with emphasis on nitrogen nutrition of crops. Effects of nitrate and ammonium nutrition on plant metabolism, development, and growth receive attention. Interactions of nitrogen with potassium and calcium are studied. Interests in composts, soil fertility, and phytoremediation are based on the effects of nitrogen on soil chemistry and plant growth.
